# Disseminated tuberculosis during TNF-α inhibitor therapy diagnosed by positron emission tomography and mini-laparoscopy

**DOI:** 10.1007/s15010-023-01979-z

**Published:** 2023-01-12

**Authors:** Lennart Hermanussen, Thomas Theo Brehm, Stefan Schmiedel

**Affiliations:** 1 Devision of Infectious Diseases, I. Department of Internal Medicine, University Medical Center Hamburg-Eppendorf, Martinistraße 52, 20246 Hamburg, Germany; 2 German Center for Infection Research (DZIF), Partner Site Hamburg-Lübeck-Borstel-Riems, Hamburg, Germany

A 40-year-old man presented to the emergency department with diffuse abdominal pain, cough, and fever. He had previously been diagnosed with ulcerative colitis, which was treated with the TNF-α inhibitor infliximab. He stated that he had lived in South Africa and Tajikistan for longer time periods. Since initial clinical and laboratory diagnostics, as well as other imaging procedures were inconclusive, a positron emission tomography (PET) was performed on suspicion of a malignancy. This showed a 18F-Fluordesoxyglucose (FDG) enrichment along the entire peritoneum as well as in mediastinal and abdominal lymph nodes (Fig. 1). For further workup, sputum samples were examined and a mini-laparoscopy with collection of peritoneal tissue samples was performed [1]. *Mycobacterium tuberculosis* was detected by PCR from the peritoneum and by mycobacterial culture from sputum samples. Tuberculostatic medication with rifampicin, isoniazid, ethambutol, and pyrazinamide was initiated and a good clinical response was observed. In patients with febrile illness during therapy with TNF-α inhibitors, the presence of extrapulmonary tuberculosis should also be considered. As was the procedure in this case, an interferon-gamma release assay (IGRA) must always be performed before initiating such therapy because of the high risk of activating a latent tuberculosis. Likewise, adults should only be treated for latent tuberculosis at an increased lifetime risk of activation [2]. However, in the setting of unclear abdominal symptoms, active extrapulmonary tuberculosis should be considered as differential diagnosis, even if the IGRA is negative [3]. PET and mini-laparoscopy could contribute crucially to the rapid diagnosis of disseminated tuberculosis in this case [4].

**Fig. 1:**
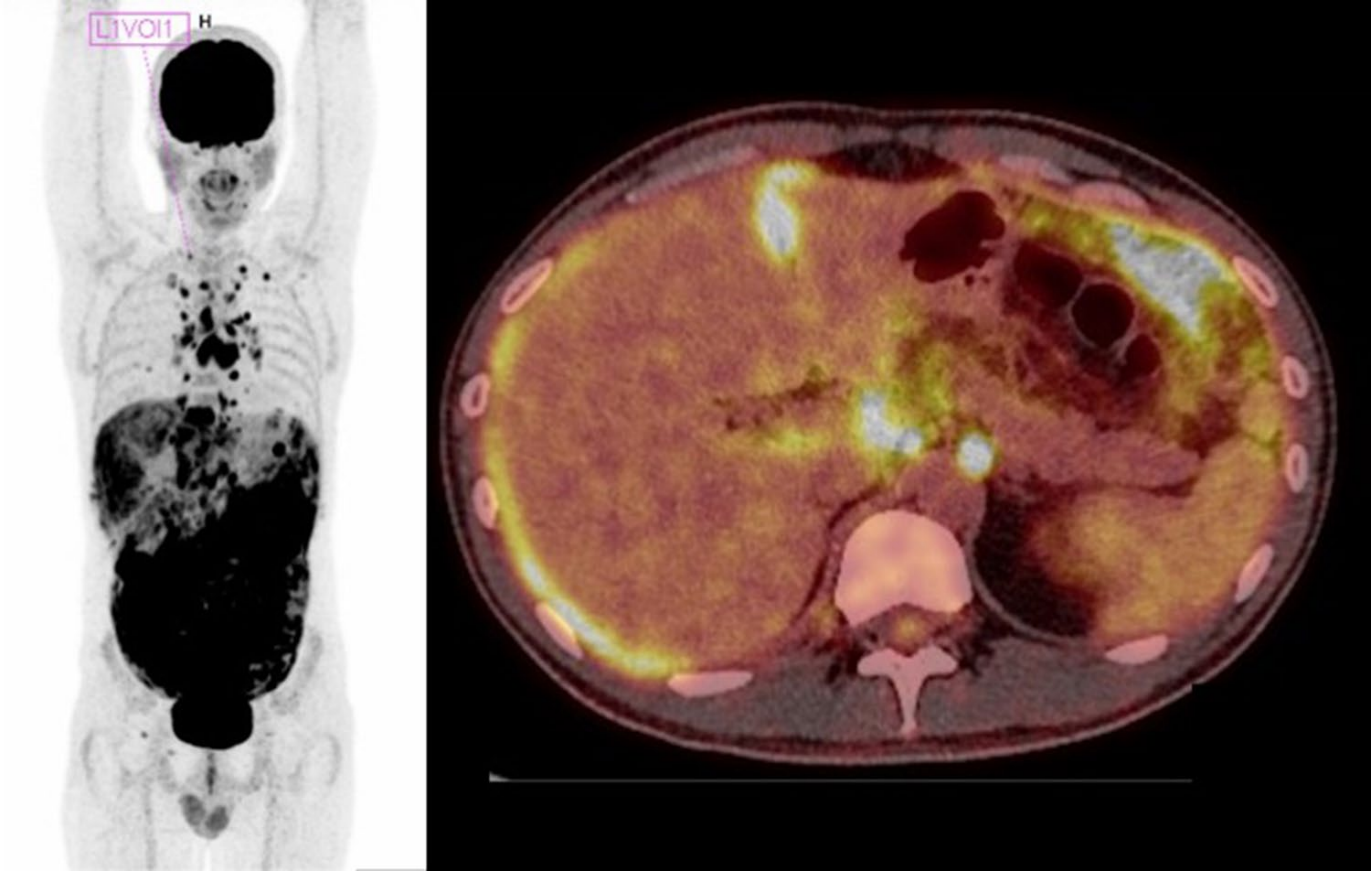
Positron emission tomography (PET) with enrichment along the peritoneum and in mediastinal and abdominal lymph nodes

## Data Availability

The data and materials are available from the corresponding author, LH, upon reasonable request.
